# MicroRNA Expression Analysis and Biological Pathways in Chemoresistant Non-Small Cell Lung Cancer †

**DOI:** 10.3390/cancers17152504

**Published:** 2025-07-29

**Authors:** Chara Papadaki, Maria Mortoglou, Aristeidis E. Boukouris, Krystallia Gourlia, Maria Markaki, Eleni Lagoudaki, Anastasios Koutsopoulos, Ioannis Tsamardinos, Dimitrios Mavroudis, Sofia Agelaki

**Affiliations:** 1 Laboratory of Translational Oncology, School of Medicine, University of Crete, 71500 Heraklion, Greece; chapapadak@uoc.gr (C.P.); mmortoglou@med.uoc.gr (M.M.); arisbouk@gmail.com (A.E.B.); krysgourlia@gmail.com (K.G.); mavroudis@uoc.gr (D.M.); 2 Department of Medical Oncology, University General Hospital, 71500 Heraklion, Greece; 3 Department of Computer Science, University of Crete, 71500 Heraklion, Greece; maria.g.markaki@gmail.com (M.M.); tsamard.it@gmail.com (I.T.); 4 Department of Pathology, University General Hospital of Heraklion, 71500 Heraklion, Greece; elagoudakimd@gmail.com (E.L.); akoutsop@gmail.com (A.K.); 5 School of Medicine, University of Crete, 70013 Heraklion, Greece

**Keywords:** non-small cell lung cancer, cisplatin, chemoresistance, miRNAs, bioinformatics

## Abstract

**Background/Objectives:** Alterations in DNA damage repair mechanisms can impair the therapeutic effectiveness of cisplatin. MicroRNAs (miRNAs), key regulators of DNA damage repair processes, have been proposed as promising biomarkers for predicting the response to platinum-based chemotherapy (CT) in non-small cell lung cancer (NSCLC). In this study, by using a bioinformatics approach, we identified six miRNAs, which were differentially expressed (DE) between NSCLC patients characterized as responders and non-responders to platinum-based CT. We further validated the differential expression of the selected miRNAs on tumor and matched normal tissues from patients with resected NSCLC. **Methods:** Two miRNA microarray expression datasets were retrieved from the Gene Expression Omnibus (GEO) repository, comprising a total of 69 NSCLC patients (N = 69) treated with CT and annotated data from their response to treatment. Differential expression analysis was performed using the Linear Models for Microarray Analysis (Limma) package in R to identify DE miRNAs between responders (N = 33) and non-responders (N = 36). Quantitative real-time PCR (qRT-PCR) was used to assess miRNA expression levels in clinical tissue samples (N = 20). **Results:** Analysis with the Limma package revealed 112 DE miRNAs between responders and non-responders. A random-effects meta-analysis further identified 24 miRNAs that were consistently up- or downregulated in at least two studies. Survival analysis using the Kaplan–Meier plotter (KM plotter) indicated that 22 of these miRNAs showed significant associations with prognosis in NSCLC. Functional and pathway enrichment analysis revealed that several of the identified miRNAs were linked to key pathways implicated in DNA damage repair, including the p53, Hippo, PI3K and TGF-β signaling pathways. We finally distinguished a six-miRNA signature consisting of miR-26a, miR-29c, miR-34a, miR-30e-5p, miR-30e-3p and miR-497, which were downregulated in non-responders and are involved in at least three DNA damage repair pathways. Comparative expression analysis on tumor and matched normal tissues from surgically treated NSCLC patients confirmed their differential expression in clinical samples. **Conclusions:** In summary, we identified a signature of six miRNAs that are suppressed in NSCLC and may serve as a predictor of cisplatin response in NSCLC.

## Simple Summary

Platinum-based chemotherapy (CT) remains the cornerstone for treatment of lung cancer; nevertheless, its effectiveness is often limited by the emergence of chemoresistance. MicroRNAs (miRNAs) have emerged as promising biomarkers for predicting treatment response in non-small cell lung cancer (NSCLC). In this study, we applied a bioinformatics approach to identify differentially expressed (DE) miRNAs linked to the response to platinum-based CT in NSCLC. We distinguished six DE miRNAs that regulate key signaling pathways implicated in cancer progression and drug resistance. Interestingly, five of these miRNAs were also downregulated in tumor tissues from NSCLC patients as compared to paired normal tissues. Overall, we identified a six-miRNA signature involved in the pathogenesis of NSCLC and with a potential predictive role for cisplatin response in NSCLC, which warrants further clinical validation.

## 1. Introduction

Lung cancer is the leading cause of cancer-related deaths in both men and women worldwide with non-small cell lung cancer (NSCLC) representing approximately 85% of all cases [1]. Although considerable progress has been made in systemic therapies, including targeted treatments and immune checkpoint inhibitors (ICIs), platinum-based chemotherapy remains the standard of care for both early and advanced NSCLC. Nonetheless, its effectiveness can be compromised by inherent or acquired resistance mechanisms within tumor cells. These include decreased intracellular drug accumulation, increased detoxification systems and aberrant DNA damage repair systems [2].

Platinum-based therapies, such as cisplatin, exert cytotoxic effects by forming intra-strand and inter-strand crosslinks, which stall the replication fork and increase toxicity in proliferating cells. Prevention of the replication fork activates the DNA damage response (DDR), followed by DNA damage repair, a complex signaling network responsible for sensing and repairing DNA lesions [3]. DNA damage repair includes several pathways, such as nucleotide excision repair (NER), mismatch repair, homologous recombination, non-homologous end joining (NHEJ), base excision repair (BER), DNA strand break repair and Fanconi anemia proteins (FA) [3]. The efficiency of the DNA damage response and repair pathways plays a central role in determining cisplatin sensitivity, while mutations or altered expressions of DNA repair genes are linked to increased platinum-based treatment sensitivity. In addition, impaired DNA repair mechanisms contribute fundamentally to cancer initiation and progression by allowing the accumulation of genetic mutations, promoting genomic instability and disabling key tumor suppressor pathways, leading to increased cell proliferation and reduced apoptosis [3]. The complexity of DNA damage repair poses significant challenges in the field of research for predictive biomarkers, as DNA damage repair pathways function as a complex signaling network that connects DNA damage repair to other signaling pathways, such as those of cell proliferation and apoptosis [3]. Thus, identifying reliable biomarkers for platinum responsiveness remains a significant challenge, aiming to prevent overtreatment in patients unlikely to benefit from the therapy.

MicroRNAs (miRNAs) are a class of small, non-coding RNA molecules, typically 20–22 nucleotides in length, that can regulate gene expression at the post-transcriptional level [4,5]. Previous studies have demonstrated that their expression can be altered in several cancer types [6]. MiRNAs can function either as oncogenes or tumor suppressor genes in critical biological processes such as tumor progression, metastasis and response to cytotoxic therapies, including cisplatin [6]. A growing body of evidence suggest that miRNAs can modulate the expression of DNA damage repair-related proteins at the post-transcriptional level in response to platinum-induced damage. Conversely, platinum-induced DNA damage alters miRNA expression through transcriptional regulation [7]. In the past few years, miRNAs have emerged as promising prognostic and predictive biomarkers in cancer [8]. Notably, bioinformatic analysis of high-throughput (“omics”) data enables the detection of miRNAs with potential predictive value in treatment response, which can subsequently be validated in clinical settings [9]. The continuous growth of available biomedical data highlights the importance of incorporating bioinformatics pipelines to gain deeper insights into the molecular mechanisms under study. In this study, we applied advanced bioinformatics approaches to determine miRNAs with differential expression related to platinum-based chemotherapy response in NSCLC. We identified six miRNAs that were differentially expressed (DE) among patients with NSCLC treated with platinum-based chemotherapy and exhibited response or non-response (resistance) to treatment. We next evaluated miRNA expression in paired tumor and normal tissue samples by using quantitative real-time PCR (qRT-PCR). Unbiased pathway enrichment analysis revealed that the identified miRNAs are involved in key signaling pathways in NSCLC. Our data warrant future validation regarding the predictive role of the above signature in NSCLC patients treated with platinum-based chemotherapy.

## 2. Materials and Methods

### 2.1. Dataset Collection

Two miRNA expression microarray datasets (GSE56036 [10], GSE56264 [11]) were obtained from the Gene Expression Omnibus Datasets (GEO; https://www.ncbi.nlm.nih.gov/geo/ (accessed on 1 October 2020). The inclusion criteria required datasets to contain NSCLC patients treated with CT along with documented treatment response data. GEO was searched using the keywords “tissue”, “NSCLC”, “platinum-based chemotherapy”, “response” and “miRNA profiling”. In total, the two datasets comprised 69 samples including 33 responders and 36 non-responders (Table 1).

### 2.2. Dataset Statistical Analysis

DE miRNAs between responders and non-responders in the two datasets were identified using the Limma package (version 3.44.3) in R software (version 3.11) [12] (https://bioconductor.org/packages/release/bioc/html/limma.html (accessed on 15 October 2020), which is designed for statistical computing and visualization. The Benjamini–Hochberg false discovery rate method was used to obtain adjusted *p*-values and to correct the occurrence of false positives. Adjusted *p*-values < 0.05 and |log fold change (logFC)| values ≥ 0.5 were set as the threshold for identifying DE miRNAs among the two datasets. Only miRNAs showing consistent upregulation or downregulation across both datasets and had |logFC|≥ 0.5 were classified as DE between responders and non-responders.

### 2.3. Survival Analysis with Kaplan–Meier (KM) Plotter

The prognostic relevance of the DE miRNAs was further evaluated using KM plotter, (https://kmplot.com/analysis/ (accessed on 25 October 2020)), an online platform that performs univariate and multivariate Cox regression analysis based on data from the Cancer Genome Atlas (TCGA) and GEO [13]. KM plotter provides overall survival (OS) data from 504 NSCLC patients with adenocarcinoma (ADC) and 472 NSCLC patients with squamous cell carcinoma (SqCC). The Hazard Ratio (HR) and long rank *p*-value for the 24 DE miRNAs were detected from meta-analysis. We selected the DE miRNAs with HR values consistent with the logFC value. Specifically, miRNAs with positive logFC were expected to have HR > 1, while those with negative logFC were expected to have HR < 1. Statistical significance was set at *p* < 0.05.

### 2.4. Pathway Enrichment Analysis by DIANA Tools

DIANA-mirPath (v3.0) (http://diana.imis.athena-innovation.gr/ (accessed on 30 October 2020)) is an advanced web-based platform for miRNA pathway analysis, offering accurate statistical evaluation, while being able to accommodate advanced pipelines [14]. MirPath integrates miRNA target predictions (CDS or 3′-UTR regions) from the DIANA-microT-CDS algorithm or even experimentally validated miRNA interactions derived from DIANA-TarBase. In this study, the DIANA-miRPath tool was used to determine the Kyoto Encyclopedia of Genes and Genomes (KEGG) pathways enriched with genes targeted by the DE miRNAs. For the visualization of the target genes, the results of the DIANA-miRPath analysis were exported and imported into Cytoscape (v3.10.3), an open-source software for network analysis and visualization [15]. A network was constructed, where nodes represent target genes and miRNAs, and edges signify interactions between miRNAs and genes.

### 2.5. Expression Analysis of the Six-miRNA Signature in Formalin Fixed Paraffin Embedded Tissues (FFPE)

#### 2.5.1. Patient Samples

Samples from 20 consecutive patients (N = 20) with resected NSCLC (adenocarcinoma subtype) referred in 2010 to the Department of Medical Oncology, University General Hospital of Heraklion, Crete, were included. Tumor and matched normal tissue samples were analyzed. Inclusion criteria for this study required that none of the patients had received chemotherapy or radiation therapy prior to surgery. Archival FFPE tissue samples were retrieved from the Department of Pathology, University General Hospital of Heraklion, Crete, and had been independently reviewed by two pathologists. Patients’ clinicopathological characteristics including age, gender, stage, grade and lymph nodes status are depicted in Table S1. All patients provided a signed informed consent to participate in the study, which was approved by the Ethics and Scientific Committee of the University Hospital of Heraklion (ID 7696; Crete, Greece).

#### 2.5.2. Total RNA Isolation from FFPE Tissue Samples

All paraffin-embedded tumors were independently evaluated by two pathologists to verify specimen validity and select the most suitable tumoral area for microdissection. Matched normal tissues were also examined. From each selective representative paraffin block, serial sections with a thickness of 5 μm were prepared and then stained with hematoxylin–eosin. Malignant cells were isolated using a piezoelectric microdissector (Eppendorf). Ten thousand cells from each sample were lysed overnight at 56°C in 400 μL lysis buffer containing 1.5 mg/mL proteinase K [16]. On the following day, RNA was extracted from the lysate using TRIZOL Reagent (Ambion, Austin, TX, USA; Life Technologies, Carlsbad, CA, USA) and twenty-five fmoles of the synthetic *C. elegans* miRNA cel-miR-39 (Qiagen Inc., Germantown, MD, USA) was added post-denaturation to each sample as an exogenous control [17]. The RNA pellet was resuspended in 30 μL of RNAse-free water, and DNase was added to eliminate genomic contamination according to manufacturers’ instructions (Thermo Scientific, Waltham, MA USA). RNA purity and quantity was assessed with a NanoDrop-1000 Spectrophotometer (Thermo Fisher Scientific, Waltham, MA, USA). All RNA samples were stored at −80 °C until further analysis.

#### 2.5.3. qRT-PCR Analysis and miRNA Expression

cDNA synthesis and qRT-PCR were performed according to manufacturers’ instructions using TaqMan technology, as previously described [18]. In brief, 20 ng of total RNA was reverse transcribed using the TaqMan miRNA Reverse Transcription kit and miRNA specific stem-loop primers (Applied Biosystems, Foster City, CA, USA; Table S2) in a 5 μL-reaction. The qRT-PCR reaction was carried out on a ViiA 7 Real-Time PCR System (Applied Biosystems, Foster City, CA, USA). The average expression level for each miRNA was determined using the 2^−ΔCt^ method, normalized to the reference gene miR-1228-3p [19,20]. Although miR-1228 has been suggested as a reference gene in the assessment of circulating miRNAs, we herein show its suitability as a reference gene in FFPE tissue samples. Specifically, (i) no changes in miR-1228 expression were observed among tumor and matched normal tissue samples (Wilcoxon test, *p* = 0.142) and (ii) it demonstrated a similar range of expression with target miRNAs since the ΔCt (ΔCt = Ct target −Ct reference) was low.

#### 2.5.4. Statistical Analysis

SPSS software version 22.0 (SPSS Inc., Chicago, IL, USA, accessed on 14 April 2022) and GraphPad Prism version 9.3.1 (GraphPad software, Inc., La Jolla, CA, USA) were used for the statistical analysis. The non-parametric Wilcoxon paired sample test was utilized to assess the differential expression of miRNAs among tumor and the corresponding normal tissue. For the evaluation of the diagnostic capability of each miRNA, receiver operating curves (ROC) were generated and the area under the curve (AUC) was calculated. The optimal cut-off value for each miRNA was determined based on the maximum Youden’s index (sensitivity + specificity − 1), and corresponding sensitivity and specificity values were reported. Statistical significance was set at *p* < 0.05 (two-sided test).

## 3. Results

### 3.1. Dataset Selection and Expression Profiling Data Analysis

**Figure 1:**
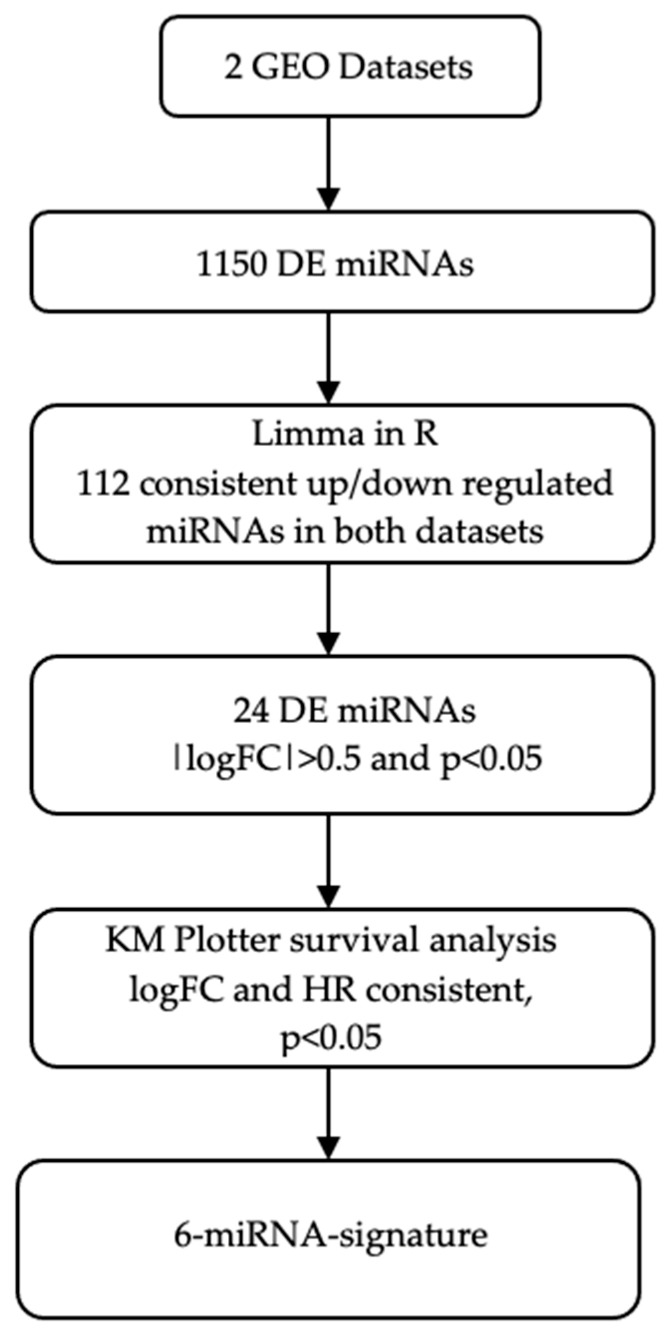
Schematic representation of the study workflow. DE, differentially expressed; GEO, gene expression omnibus; HR, hazard ratio; KM plotter, Kaplan–Meier plotter; logFC, logarithm of fold change.

Figure 1 illustrates the workflow of the current study. Two GEO datasets comprising 69 NSCLC patients (responders/non-responders) treated with platinum-based CT (Table 1) were analyzed. Initial analysis using the Limma R package (3.64.1) identified 1614 DE miRNAs and the number was decreased to 1150 miRNAs, after excluding miRNAs with zero expression values. Meta-analysis revealed 72 consistently upregulated and 40 downregulated DE miRNAs across both datasets (Figure 1). Furthermore, out of the 112 DE miRNAs, 24 were up- or downregulated with |logFC| ≥ 0.5 and *p*-value < 0.05.

### 3.2. Survival Analysis by KM Plotter

**Figure 2:**
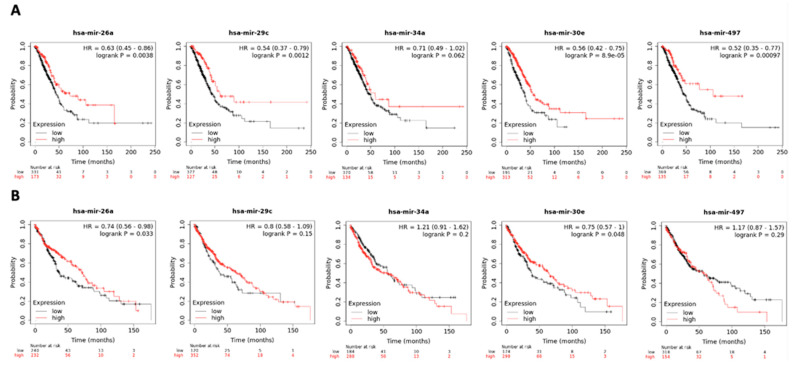
Survival analysis of hsa-miR-26a, hsa-miR-29c, hsa-miR-34a, hsa-miR-30e and hsa-miR-497 in (**A**) adenocarcinoma (N = 504) and (**B**) squamous cell carcinoma (N = 472) in KM plotter dataset. Samples are categorized as high (red) and low (black) expression groups for each miRNA. HR and *p*-value for each miRNA associated with survival are shown within the respective plot.

As a validation step, KM plotter was used to evaluate the prognostic value of the 24 DE miRNAs by assessing their association with OS in patients with NSCLC. We examined the concordance of the logFC as reported by Limma with the corresponding HR provided by KM plotter and further identified the DE miRNAs, which were consistently up- or downregulated in NSCLC patients (Table 2 and Figure 2). Therefore, this last integration resulted in a six-miRNA signature, comprising hsa-miR-26a, hsa-miR-29c, hsa-miR-30e-5p, hsa-miR-30e-3p, hsa-miR-34a and hsa-miR-497. Of those, only miR-34a was non-significantly associated with OS.

### 3.3. Validation of the Predicted Six-miRNA Signature by Pathway Enrichment Analysis

**Figure 3:**
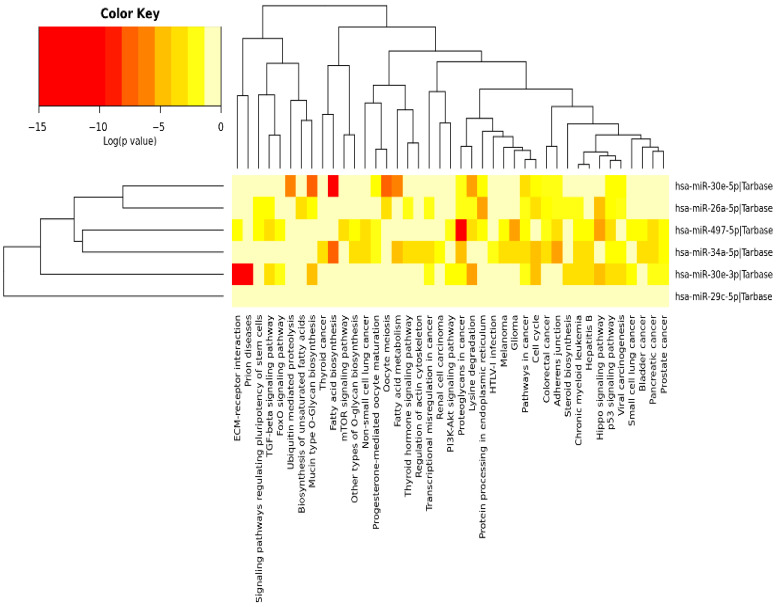
Heatmap illustrating the pathways significantly enriched among the targets of the 6 miRNAs. DIANA-miRPath (v3.0) was used to perform the pathway enrichment analysis identifying significantly enriched KEGG pathways associated with the DE miRNAs. Color key shows the statistical significance expressed by the log (*p*-value); as the color becomes darker, the involvement of the specific miRNA becomes more statistically significant in the specific pathway [14].

**Figure 4:**
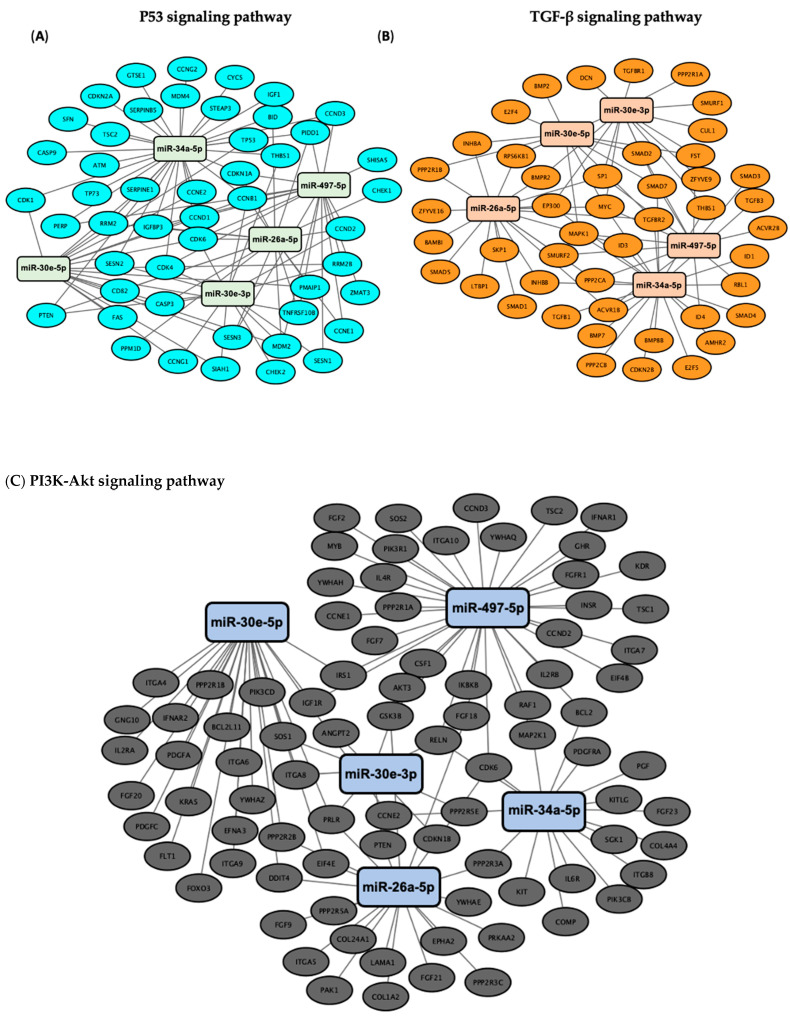

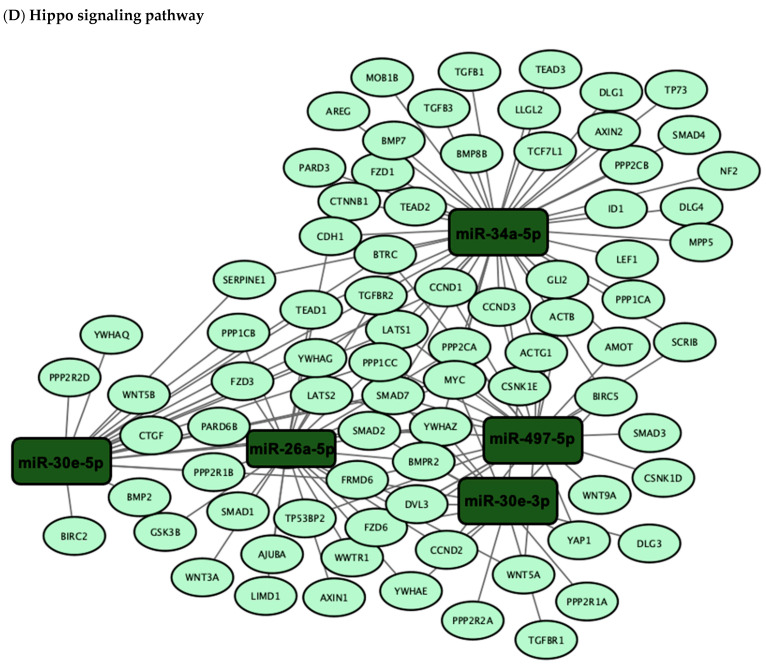
Network visualization of target genes associated with DE miRNAs in the (**A**) p53, (**B**) TGF-β, (**C**) PI3K-Akt and (**D**) Hippo signaling pathways. The network visualization represents the results of pathway enrichment analysis conducted using DIANA-miRPath (v3.0) to identify target genes associated with the six-miRNA signature. DE miRNAs and their target genes involved in the p53, TGF-β, PI3K-Akt and Hippo signaling pathways were selected and visualized using Cytoscape (v3.10.3). In the network, edges represent the miRNA–gene interactions.

Pathway enrichment analysis for the six miRNAs identified significantly enriched cancer-associated pathways, including “cancer-related pathways”, “apoptosis and proliferation”, “proteoglycans in cancer”, as well as the p53, Hippo, TGF-β, insulin and PI3K-Akt signaling pathways (Figure 3). Among those, the p53, Hippo, TGF-β and PI3K-Akt signaling pathways have been previously implicated in DNA damage repair [21,22,23,24], a key regulator of cellular sensitivity and resistance to cisplatin; therefore, we further identified how their target genes correlated to the DE miRNAs. Specifically, we utilized Cytoscape (v3.10.3) (https://cytoscape.org/) to visualize the DIANA-miRPath analysis results (Figure 4). All of the identified miRNAs, other than hsa-miR-29c, show significant involvement in at least two of the aforementioned pathways. miR-29c potentially acts through alternative mechanisms not highlighted by our pathway analysis. This may reflect its involvement in regulating a diverse set of targets across multiple pathways involved in cisplatin resistance, such as apoptosis and epithelial-to-mesenchymal transition (EMT), which may not have reached statistical significance in our pathway enrichment analysis [25].

### 3.4. Evaluation of Six-miRNA Signature in Paired Normal and Tumor FFPE NSCLC Tissues

**Figure 5:**
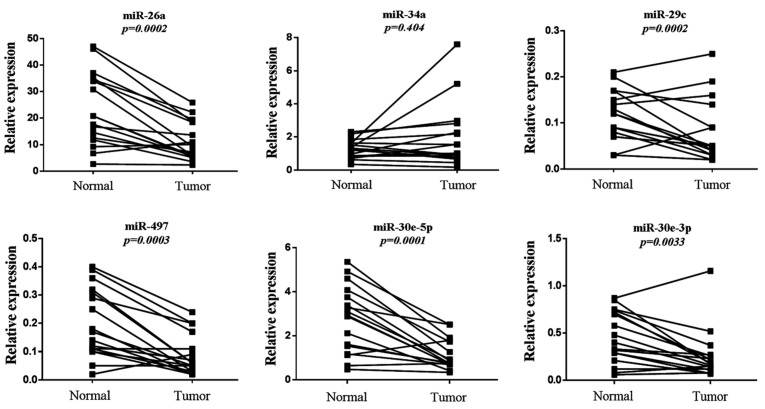
Relative expression levels of the six-miRNA signature in primary tumors and paired normal tissues. Comparison for each miRNA was performed by non-parametric Wilcoxon paired sample test. The Y-axis denotes expression levels for each miRNA relative to miR-1228 assessed by 2^−ΔCT^.

**Figure 6:**
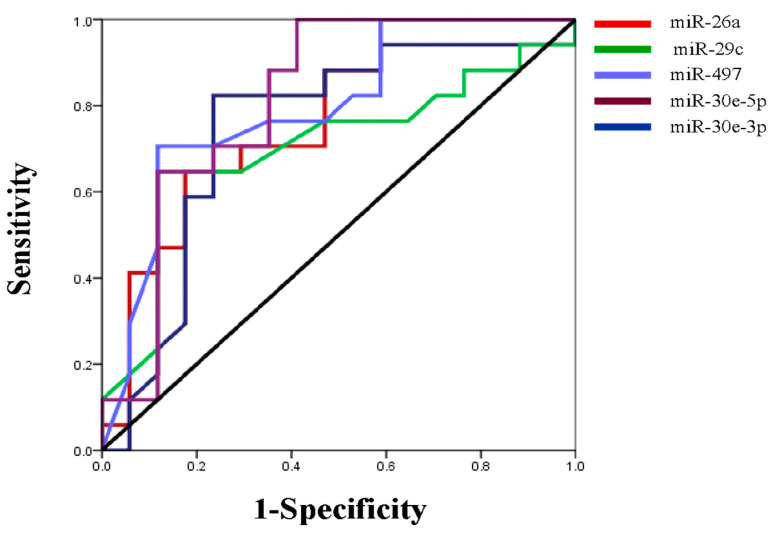
ROC curve was generated to evaluate the potential of miRNAs as a biomarker to discriminate primary tumor from normal tissues.

We next evaluated the expression profile of the six-miRNA signature in primary lung adenocarcinoma and their paired normal tissues by qRT-PCR. All miRNAs were significantly downregulated in the tumor compared to normal tissues, except for miR-34a, the same miRNA that showed the weakest prognostic value in our KM plotter analysis (Figure 5). Specifically, the expression of miR-26a, miR-29c, miR-30e-5p, miR-30e-3p and miR-497 were more than two-fold higher in normal tissue compared to primary tumor tissue (Table 3). We further performed ROC curve analysis to evaluate their diagnostic capability. miR-30e-5p along with miR-497 had the highest power to discriminate primary tumors from normal tissues with an AUC of 0.813 (95% CI: 0.661-0.966; *p* = 0.002 with a sensitivity of 88.2% and a specificity of 64.7%) and 0.794 (95% CI: 0.639–0.966; *p* = 0.003 with a sensitivity of 70.7% and a specificity of 88.2%), respectively (Table 4, Figure 6).

## 4. Discussion

Although platinum-based chemotherapy is the standard-of-care for both early-stage and advanced NSCLC cases, its efficacy is often limited by the development of primary or acquired resistance [2]. Enhancing prognostic tools and developing personalized treatment strategies remains a critical challenge in improving patient outcomes. In recent years, miRNAs have emerged as promising biomarkers for diagnosis, prognosis and prediction of treatment responses [26], while also being recognized as key drivers of chemoresistance by modulating pathways involved in drug efflux, apoptosis evasion and DNA damage repair [27]. In the present study, by applying a multistep bioinformatics approach, we identified a six-miRNA signature (miR-26a, miR-29c, miR-497, miR-34a, miR-30e-3p, miR-30e-5p) that was differentially expressed between patients with NSCLC characterized as responders and non-responders to cisplatin-based therapy, and which was also decreased in NSCLC tumors compared to paired normal tissues. Previous studies have also proposed miRNA-based signatures for predicting chemotherapy response in NSCLC. For instance, a three-miRNA signature (miR-21, miR-125b and miR-224) has been suggested as a predictor of the response to platinum-based chemotherapy in lung adenocarcinoma [28]. However, while these studies have demonstrated prognostic value, they often lack integrative pathway enrichment analysis, in contrast to our study.

miRNA expression profiling studies on tissues have identified the altered expression of miRNAs in lung cancer associated with diagnosis, staging, progression, prognosis and response to treatment [10,11,29]. Notably, miR-26a is downregulated in several cancer types and acts as a tumor suppressor [7,30], while miR-26a loss is associated with tumor progression [31]. Importantly also, miR-26a plays a significant role in the regulation of apoptosis in various cancer types, including lung cancer. Results from a recent study demonstrate that miR-26a induces cell apoptosis and inhibits autophagy by targeting the TGF-β-JNK signaling pathway [31]. In another report, miR-26a was shown to sensitize NSCLC cells to chemotherapy through the regulation of apoptosis-related proteins. In addition, the restoration of miR-26a expression increased cisplatin activity by targeting the HMGA2/E2F/Akt signaling pathway and suppressing Bcl-2. This regulation leads to cell cycle arrest, enhanced apoptosis and decreased colony formation [7]. Moreover, miR-26a may also contribute to increased metastatic potential in lung cancer cells by suppressing *PTEN* expression via activation of the AKT pathway [32]. Similarly, the expression of miR-29 family members (miR-29a, miR-29b and miR-29c) is downregulated in several malignancies, including lung cancer, where they function as tumor suppressors [33]. The miR-29 family directly targets the two key enzymes of DNA methylation, DNA methyltransferase (DNMT) 3A and 3B, and restores normal patterns of DNA methylation [34]. Of particular interest, miR-29c suppresses migration and invasion by directly targeting VEGF, whereas its downregulation is significantly related to unfavorable prognosis in lung cancer [35]. Furthermore, it has been previously demonstrated that the enforced expression of miR-29c enhances cisplatin sensitivity in lung cancer cells by targeting AKT2, while the silencing of miR-29c promotes cisplatin resistance in cancer cells by activating the PI3K/Akt pathway. This regulation leads to reduced cell viability upon cisplatin treatment, suggesting that miR-29c moderation could be a potential therapeutic target to overcome cisplatin resistance in NSCLC [33]. In accordance with these findings, we also found that miR-26a and miR-29c are both downregulated in lung cancer compared to the corresponding normal tissues, underscoring their potential roles in tumor suppression and treatment response.

The tumor-suppressive roles of miR-34a and miR-497 have also been previously highlighted, with evidence pointing to the regulation of cell cycle progression and the promotion of apoptosis [36,37]. Specifically, miR-34a is transcriptionally regulated by p53 in response to DNA damage and oncogenic stress, contributing to p53-mediated apoptosis [38]. Additionally, miR-34a promotes G1 arrest by targeting cell cycle-related proteins, including Cyclin D1 and CDK4/6 [39]. It has also been reported that miR-34a along with miR-497 inhibit lung cancer cell growth by cooperatively regulating the expression of cyclin E1 [39]. Moreover, in colon cancer, miR-34a has been shown to inhibit the expression of numerous ATP-binding cassette (ABC) transporters such as MRP2, P-gp and BCRP, as well as antiapoptotic genes like *Bcl-2*, all of which contribute to chemoresistance. Particularly, ABC transporters can actively expel intracellular chemotherapeutic drugs, including oxaliplatin, significantly reducing their efficacy, while the overexpression of *Bcl-2* was shown to protect colon cancer cells from chemotherapy-induced apoptosis, further promoting oxaliplatin resistance [40]. miR-34a and miR-497 are located on chromosomes 1p36 and 17p13.1, respectively, sites that are frequently lost or deleted in several cancer types including lung cancer [41]. miR-497 has been described as a tumor suppressor and, similar to miR-34a, is downregulated in lung cancer tissues and cells [42,43]. Importantly, miR-34a modulates the p53/miR-34a/MYCN pathway to enhance the effectiveness of cisplatin therapy in lung cancer [6], whereas miR-497-5p enhances the cisplatin sensitivity of lung cancer cells by inhibiting Yes-associated protein 1 (YAP1) and TEA domain family member 1 (TEAD1), key components of the Hippo pathway [42]. These findings suggest that the downregulation of both miR-34a and miR-497 not only promotes tumor progression but also may contribute to chemoresistance by enabling apoptotic evasion and/or drug efflux [6,42,43]. Although miR-34a is commonly reported as downregulated in NSCLC, our cohort showed only a slight, non-significant increase in tumor tissues compared to normal. This inconsistency may reflect biological heterogeneity or, perhaps, a limitation of our small sample size, highlighting the need for further investigation in larger patient cohorts to better elucidate the role of miR-34a in NSCLC. However, in our study, miR-497 was differentially expressed among tumor and normal tissues, warranting the further evaluation of its clinical relevance.

Additionally, miR-30e, a member of the miR-30 family (miR-30a/b/c/d/e), has also been implicated in NSCLC progression, with prior studies demonstrating its tumor-suppressive functions [44]. Particularly, miR-30e-5p has been shown to inhibit cell proliferation and invasion in NSCLC by directly targeting SOX9, a transcription factor involved in tumorigenesis and metastasis [45]. Furthermore, miR-30e-5p reduces tumorigenesis by targeting Sirt1/JAK/STAT3 signaling, which plays a critical role in cancer cell survival, proliferation and immune evasion [46]. Specifically, growing evidence indicates a potential role of the miR-30 family in regulating DNA damage repair and chemoresistance mechanisms. For example, in ovarian cancer, a feedback loop between miR-30a/c-5p and DNMT1 has been identified as a key regulator of cisplatin resistance and EMT, processes intimately linked to DNA damage repair signaling [47]. In accordance, our results showed that miR-30e is downregulated in primary lung cancer tumors as compared to normal tissues, implying that its reduced expression may be implicated in tumor progression, whereas its role in cisplatin resistance requires further investigation using clinical samples.

Our KEGG pathway enrichment analysis revealed that the DE miRNAs were involved in key cancer-related pathways, such as cell cycle, proteoglycans in cancer, focal adhesions, Hippo signaling, p53 signaling, TGF-β signaling, insulin signaling and PI3K-Akt signaling, many of which participate in the regulation of DNA damage repair [22,23,48,49], one of the primary mechanisms of resistance against cisplatin-induced cytotoxicity [50]. Therefore, we focused our pathway enrichment analysis on DNA damage repair-related pathways to further elucidate the functional roles of the DE miRNAs in our signature. Among the identified pathways, the p53 signaling plays one of the most critical roles in maintaining genomic stability and regulating key cellular processes such as cell cycle arrest, apoptosis and DNA repair. Specifically, DDR triggered by different p53 isoforms can either promote the canonical p53 DNA damage repair or inhibit cell death pathways potentially leading to chemoresistance [51]. Mutations in *TP53* have been identified in approximately 50% of NSCLC patients and are associated with poor prognosis and resistance to therapy [52,53]. Moreover, alterations in critical DNA damage repair genes including *TP53* have been linked to cancer progression and metastasis, underscoring their significance in NSCLC development [53]. Importantly also, recent findings indicate that cisplatin sensitivity in NSCLC cells can be modulated through the p53/miR-34a/MYCN signaling pathway [6].

In addition to p53-related pathways, other signaling cascades, such as the Hippo pathway, play a crucial role in cancer by regulating key cellular processes. Dysregulation of this pathway is commonly observed across various human malignancies [54]. YAP and the transcriptional co-activator with PDZ-binding motif (TAZ), key downstream mediators of the Hippo pathway, are increasingly recognized for their roles in regulating DNA damage repair and contributing to cisplatin resistance. A recent study showed that the amplification frequency of YAP and TAZ across 33 cancer types ranges from 0-19%, with lung squamous cell carcinoma exhibiting the second-highest frequency [55]. Additionally, YAP shows greater nuclear enrichment in NSCLC compared to healthy tissues [56], while elevated expression levels of YAP and TAZ have been linked to poorer survival outcomes in NSCLC patients [57]. Previously, it has been proposed that upon DNA damage, YAP can be activated in a p53-dependent or p53-independent manner, promoting transcriptional programs that facilitate cell survival, proliferation and repair [58]. Furthermore, cells with elevated YAP and TAZ expression can overcome the DNA damage and the inhibition of DNA synthesis induced by chemotherapy, suggesting that targeting YAP and TAZ can be a promising strategy to overcome cisplatin resistance [59]. Taken together, the interplay between YAP/TAZ and DNA damage repair, particularly in the context of p53 dysregulation, represents a critical mechanism of platinum resistance in NSCLC, and underscores the biological relevance of the six-miRNA signature identified in our analysis.

Besides the p53 and Hippo pathways, the TGF-β pathway is also important for the progression and treatment resistance of NSCLC. TGF-β overexpression has been associated with tumor progression and is considered a marker of poor prognosis [60]. Furthermore, elevated TGF-β levels contribute to drug resistance by modulating the immune response, promoting tumor vascularization and reducing the effectiveness of both antiangiogenic therapies and immune checkpoint inhibitors [61]. TGF-β is also a key driver of EMT, facilitating tumor initiation, invasion and metastasis by regulating multiple signaling pathways, including MAPK/ERK1/2, NF-κB/Snail, JAK/STAT3 and PI3K/AKT signaling [62]. Interestingly, it has been previously shown that cisplatin enhances the secretion of TGF-β1, which may lead to mesenchymal-like phenotypic changes and the subsequent development of cisplatin resistance in NSCLC [63]. TGF-β signaling also directly influences DNA damage repair mechanisms in a multifaceted manner by modulating the expression of key DNA repair genes, such as *BRCA1* and *ATM* [64]. Moreover, TGF-β can enhance p53-mediated cell cycle arrest and apoptosis through the Smad-dependent transcriptional activation of p21, while p53 can repress TGF-β-induced EMT by downregulating key effectors, highlighting a bidirectional regulatory loop that influences DNA damage repair outcomes and therapy resistance [65]. Collectively, these data suggest that inhibiting this pathway may improve the effectiveness of current therapies, such as chemotherapy, targeted therapy and immunotherapy [61].

Furthermore, our KEGG analysis identified PI3K-Akt as one of the enriched pathways targeted from our six-miRNA signature. Growing evidence indicates that the PI3K/AKT/mTOR signaling pathway is frequently activated in NSCLC, playing a crucial role in tumorigenesis by promoting cell survival, growth, proliferation and migration. This pathway is heavily involved in both the initiation and progression of NSCLC, with *PIK3CA* somatic mutations and gene amplifications commonly detected in affected patients [66]. Hyperactivation of the PI3K/Akt pathway has been linked to cisplatin resistance through the regulation of the Bax-mitochondria-mediated apoptosis pathway in lung cancer [67]. Specifically, overactivation of this pathway can induce the DDR process and failure of chemo-radiotherapy with increased DNA damage repair. Moreover, PI3K-Akt signaling can enhance DNA repair through the moderation of DNA damage repair-related proteins such as BRCA1, HMGB1 and P53 [68,69]. Therefore, inhibiting the PI3K/Akt signaling pathway can sensitize these resistant cells to cisplatin, effectively reversing the resistance.

In summary, we developed a six-miRNA signature profile with tumor-suppressive properties, which have been linked to both sensitivity and resistance to various anticancer treatments, including cisplatin [5,17]. The predictive relevance of the six-miRNA signature was suggested through analysis of GEO datasets comprising NSCLC patients stratified as responders or non-responders to platinum-based chemotherapy, where these miRNAs were found to be DE between the two groups. We further examined the clinical relevance of the six-miRNA signature in a local cohort of paired tumor and normal lung tissues, where five out of six miRNAs were significantly downregulated in tumors compared to matched normal tissues, consistent with their proposed tumor-suppressive roles. ROC curve analysis revealed that all of the examined miRNAs, except for miR-34a, exhibited strong efficiency in distinguishing between tumor and normal tissues, highlighting their potential as diagnostic biomarkers. A limitation of our study is the small sample size of the datasets used in the bioinformatics analysis, which have however been previously evaluated in similar NSCLC studies, supporting their potential as biological and clinical biomarkers. Furthermore, these studies are the only publicly available datasets in GEO with annotated platinum-based chemotherapy response information and miRNA profiling in NSCLC tissue samples. To further strengthen our findings, validation in larger cohorts of NSCLC patients treated with platinum-based chemotherapy, using multivariate analysis to adjust for clinical variables, is warranted to evaluate the predictive value of the six-miRNA signature.

Collectively, our six-miRNA signature not only incorporates miRNAs with suggested prognostic significance across NSCLC tissue samples but also highlights their convergence on DNA damage repair-related signaling pathways, such as p53, Hippo, TGF-β and PI3K-Akt, offering a broader mechanistic insight into chemoresistance. Furthermore, several of these miRNAs have been reported to promote cisplatin resistance in lung cancer through additional mechanisms such as drug efflux and the suppression of apoptotic pathways. Given the lack of measurable predictive biomarkers for response to platinum therapy (in lung and other cancers), our six-miRNA signature holds promise in the efforts towards personalized treatment strategies, sparing the associated toxicity in patients unlikely to respond. Finally, our findings provide hypothesis-generating evidence for the exploration of therapeutic strategies aiming at restoring the tumor-suppressing function of the identified miRNAs in case of miRNA loss-of-function, using miRNA mimics, i.e., synthetic versions of naturally occurring miRNAs that can be exogenously administered. The miR-34a analog, MRX34, was the first one to be tested in a phase I clinical trial, in 47 patients with advanced solid tumors, including NSCLC (NCT01829971), providing a signal of efficacy [70]. Mimics for the other miRNAs of our signature (miR-26a, miR-29c, miR-497, miR-30e-3p and miR-30e-5p) are also available and have been tested in vitro and in vivo; however, their integration in clinical trials is still lacking [71,72,73,74]. Combinations of miRNA mimics with DNA-damaging agents, such as cisplatin, may offer a new platform for circumventing chemoresistance; however, the feasibility and safety of this approach needs to be thoroughly tested in proof-of-concept clinical studies.

## 5. Conclusions

In summary, our study provides valuable insights into the role of miRNAs in the biology and chemoresistance of NSCLC. By employing a bioinformatics approach, we identified six DE miRNAs that target key signaling pathways involved in cancer progression and drug resistance. These findings contribute to the understanding of the molecular mechanisms underlying the response to platinum-based chemotherapy in NSCLC. Moreover, the identified six-miRNA signature holds promise as a clinically applicable biomarker for distinguishing among favorable and poor responders to this type of therapy. Moving forward, clinical validation of this miRNA signature could enable the early identification of NSCLC patients at risk of cisplatin resistance and guide the selection of alternative therapeutic strategies, ultimately improving survival outcomes and enhancing the quality of life for patients with NSCLC.

## Figures and Tables

**Table 1:** Information about the GEO datasets used for the identification of differentially expressed miRNAs, including the number of responders and non-responders and the sample source.

Accession	Platform	Responders (N)	Non-Responders (N)	Material	PMID	Year
GSE56036	GPL15446	17	12	Frozen tissue	25597412	2015
GSE56264	GPL16770	16	24	Frozen tissue	25142144	2014

**Table 2:** LogFC and *p*-values retrieved from Limma and KM Plotter analysis for the 6 miRNAs.

DE miRNAs	GSE56036		GSE56264		ADC		SqCC	
	logFC	*p*-Value	logFC	*p*-Value	HR	*p*-Value	HR	*p*-Value
hsa-miR-26a	−1.4049	0.016655	−0.52735	0.023449	0.63	0.038	0.74	0.033
hsa-miR-29c	−1.0811	0.013295	−0.82175	0.003344	0.54	0.012	0.8	0.15
hsa-miR-30e-5p	−1.19279	0.024696			0.56	8.9 × 10^−0.5^	0.75	0.048
hsa-miR-30e-3p			−0.53971	0.043327	0.56	8.9 × 10^−0.5^	0.75	0.048
hsa-miR-34a	−1.31859	0.01225	−0.45893	0.041946	0.71	0.062	1.21	0.2
hsa-miR-497	−0.99268	0.010906	−0.82107	0.019803	0.52	0.0009	1.17	0.29

ADC, adenocarcinoma; HR, hazard ratio; KM plotter, Kaplan–Meier plotter; LogFC, log fold change; SqCC, squamous cell carcinoma.

**Table 3:** Median expression values of each miRNA and fold change of their expression among primary tumors and their corresponding normal tissues.

miRNA	Normal		Tumor			
	Expression Value	±SD	Expression Value	±SD	FC	*p*-Value
miR-26a	20.81	13.75	9.29	11.17	2.24	0.0002
miR-29c	0.12	0.054	0.05	0.067	2.4	0.0002
miR-30e-5p	2.93	1.51	0.78	0.69	3.76	0.0001
miR-30e-3p	0.4	0.27	0.17	0.26	2.35	0.0033
miR-34a	1.31	0.54	1.02	1.91	1.28	0.4040
miR-497	0.17	0.12	0.06	0.07	2.83	0.0003

FC, fold change; SD, standard deviation; expression levels relative to miR-1228 were calculated by 2^−ΔCt^ method.

**Table 4:** Performance of miRNAs to discriminate primary tumor from normal tissues.

miRNAs	Cut-Off	Sensitivity (%)	Specificity (%)	AUC (95% CI)	*p*-Value
miR-26a	11.43	64.7	82.4	0.772 (0.612–0.930)	0.007
miR-29c	0.060	64.7	88.2	0.704 (0.519–0.889)	0.042
miR-497	0.095	70.6	88.2	0.794 (0.639–0.949)	0.003
miR-30e-5p	2.020	88.2	64.7	0.813 (0.661–0.966)	0.002
miR-30e-3p	0.280	82.4	76.5	0.744 (0.564–0.924)	0.015

AUC, area under curve; CI, confidence intervals; significance was set at *p*-value < 0.05.

## Data Availability

The publicly available datasets analyzed in this study are displayed in Table S1. Patients’ data analysis will be available upon request.

## Supplementary Materials

The following supporting information can be downloaded at https://www.mdpi.com/article/10.3390/cancers17152504/s1, Table S1: Patients’ clinicopathological characteristics, Table S2: Assay ID for each miRNA used in the study.

## Abbreviations

The following abbreviations are used in this manuscript:

ABC	ATP-binding cassette
ADC	Adenocarcinoma
AUC	Area Under the Curve
BER	Base Excision Repair
CI	Confidence Intervals
CT	Platinum-based Chemotherapy
DDR	DNA Damage Response
DE	Differentially Expressed
DNMT	DNA Methyltransferase
ΕΜΤ	Εpithelial-to-Μesenchymal Τransition
FA	Fanconi Anemia Proteins
FC	Fold Change
FFPE	Formalin Fixed Paraffin Embedded Tissues
GEO	Gene Expression Omnibus
HR	Hazard Ratio
ICIs	Immune Checkpoint Inhibitors
KEGG	Kyoto Encyclopedia of Genes and Genomes
KM Plotter	Kaplan–Meier Plotter
Limma	Linear Models for Microarray Analysis
LogFC	Logarithm of Fold Change
MiRNAs	MicroRNAs
NER	Nucleotide Excision Repair
NHEJ	Non-Homologous End Joining
NSCLC	Non-Small Cell Lung Cancer
qRT-PCR	Quantitative Real-Time PCR
ROC	Receiver Operating Curves
SD	Standard Deviation
SqCC	Squamous Cell Carcinoma
TAZ	Transcriptional co-Activator with PDZ-binding motif
TCGA	The Cancer Genome Atlas
TEAD1	TEA Domain Transcription Factor 1
YAP	Yes-Associated Protein
