# Supplementary material for: MicroRNA Expression Analysis and Biological Pathways in Chemoresistant Non-Small Cell Lung Cancer
Source: Cancers (Basel). 2025 Jul 29;17(15):2504. doi: 10.3390/cancers17152504 (PMC12346453; doi:10.3390/cancers17152504)
Supplement: Supplementary file 1 [file cancers-17-02504-s001.zip › cancers-3728545-supplementary.pdf]

Table S1: Patients' clinicopathological characteristics.

	Patients	
	N=20	%
**Characteristic**		
**Age, median (range)**	66 (56-83)	
<66	10	50
≥66	10	50
**Gender**		
Male	16	80
Female	4	20
**Stage**		
I	5	25
II	9	45
IIIA	6	30
**Grade**		
1	2	10
2	8	40
3	5	25
4	5	25
**Lymph nodes**		
Negative	14	70
Positive	6	30

Table S2: Assay ID for each miRNA used in the study.

**Assay name**	**Assay ID**
hsa-miR-26a-5p	000405
hsa-miR-29c-5p	001818
hsa-miR-34a-5p	000426
hsa-miR-30e-5p	002223
hsa-miR-30e-3p	000422
hsa-miR-497-5P	001043
hsa-miR-1228-3p	002919
cel-miR-39	000200
